# AI-Powered Thermography for Diabetic Foot Risk Stratification: Multicenter Cross-Sectional Study

**DOI:** 10.2196/81289

**Published:** 2025-11-27

**Authors:** Meshari F Alwashmi, Mustafa alghali, Waseem Abu-Ashour, Abdullah Mohammed Arabe, Abdullah Al Soheimi, Nasibh Abdulrahman Alharbi, Hani Mohammed Badahdah

**Affiliations:** 1 Amplifai Health Riyadh Saudi Arabia; 2 Memorial University, Health Sciences Centre St. John's, NL Canada; 3 Ministry of Health Riyadh, null Saudi Arabia

**Keywords:** artificial intelligence, AI, digital health, thermography, diabetic foot ulcer, risk stratification, screening

## Abstract

**Background:**

Diabetic foot complications are among the most severe and costly outcomes associated with diabetes, with high prevalence particularly in the Middle East and North Africa region. Current screening tools are often limited by subjectivity, invasiveness, or scalability challenges, underscoring the need for innovative approaches.

**Objective:**

This multicenter study aimed to evaluate the performance of an artificial intelligence (AI)–powered thermographic system, Thermal Foot Scan (TFScan), in identifying patients at elevated risk of diabetic foot complications through noninvasive temperature profiling.

**Methods:**

A multicenter cross-sectional analysis of deidentified routine screening data across 4 regions in Saudi Arabia was conducted enrolling 1120 individuals with diabetes. Participants underwent thermal imaging using a smartphone-compatible infrared camera with AI algorithms analyzing angiosomal temperature patterns and asymmetries. Risk was stratified into 4 categories (very low, low, moderate, and high). Associations between TFScan classifications and clinical risk factors, symptoms of neuropathy, and thermal abnormalities were assessed.

**Results:**

While 90.7% (1016/1120) of the participants were classified as very low or low risk, 9.3% (104/1120) were identified as moderate or high risk. This higher-risk group exhibited significantly greater prevalence of key diabetic complications (*P*<.001). Peripheral artery disease was present in 20.2% (21/104) of moderate- and high-risk participants compared to just 0.8% (8/1016) of lower-risk individuals. Cardiovascular disease (60/104, 57.7% vs 313/1016, 30.8%), neuropathy (12/104, 11.5% vs 37/1016, 3.6%), foot deformities (15/104, 14.4% vs 6/1016, 0.6%), and symptoms of loss of protective sensation (53/104, 51% vs 354/1016, 34.8%) were all significantly more frequent in the high-risk subgroup than in the low-risk group, respectively. Thermal imaging further revealed pronounced abnormalities: temperature asymmetries of ≥2.2 °C were observed in 7.1% (79/1120) of the patients overall, with the highest asymmetry and thermal change index scores concentrated in the moderate- and high-risk groups. These individuals also exhibited greater deviations in angiosomal temperature differences—exceeding 2.2 °C in key vascular territories such as the medial plantar and lateral plantar arteries—suggesting both early inflammatory states and critical perfusion deficits.

**Conclusions:**

The TFScan system effectively stratified patients with diabetes into clinically meaningful risk categories, with moderate- and high-risk groups exhibiting a significantly higher burden of vascular, neuropathic, and thermal abnormalities. However, the cross-sectional design, partial reliance on self-report, and low prevalence of advanced complications may limit causal inference. These findings highlight the potential of AI-enhanced thermography to serve as a scalable, objective screening tool for proactive diabetic foot management. Further longitudinal studies are warranted to validate its predictive power and support widespread clinical adoption.

## Introduction

### The Growing Burden of Diabetic Foot Ulcers

Diabetes affects 1 in 10 adults worldwide (537 million), with higher prevalence in the Middle East and North Africa region, affecting 1 in 6 adults (73 million) [1]. Despite advances in medical therapies, the global prevalence of diabetes mellitus is projected to reach 643 million by 2030, with diabetes-related complications also continuing to rise.

One of the most common complications of diabetes is diabetic foot ulcer (DFU). It is estimated that one-third of people with diabetes will develop a DFU during their lifetime [2]. Unfortunately, even after a DFU has been resolved, recurrence is common and is estimated to be 40% within 1 year and 60% within 3 years [3]. Furthermore, diabetes foot care costs are the single largest category of diabetes-related medical costs, accounting for one-third of all diabetes-related costs, and a leading cause of lower-limb amputations [2]. Prevention of these lower-limb complications could lead to a significant positive impact on health outcomes and significant cost savings. Unfortunately, current tools for assessing the risk of developing DFUs have limited scalability in terms of time efficiency and practicality [4].

### Limitations of Existing Screening Methods

Traditional tools used to detect diabetic foot complications include the monofilament test, the ankle-brachial index, duplex ultrasonography, and angiography. While widely used, these methods have notable limitations. The monofilament test is designed specifically to assess protective sensations and is not expected to evaluate structural deformities and vascular insufficiencies, with accuracy often dependent on examiner technique [5]. The ankle-brachial index, though noninvasive, is less effective in populations with diabetes due to vessel calcification, which can lead to falsely elevated readings [6,7]. Duplex ultrasonography, while useful for detecting arterial narrowing, is limited in visualizing distal vessels in diabetic limbs [8,9]. Angiography remains the gold standard for vascular imaging but is expensive, time-consuming, and associated with radiation exposure and procedural risks [10]. These limitations highlight the need for a more sensitive, accessible, and objective screening tool.

### Human Medical Thermography

**Figure 1:**
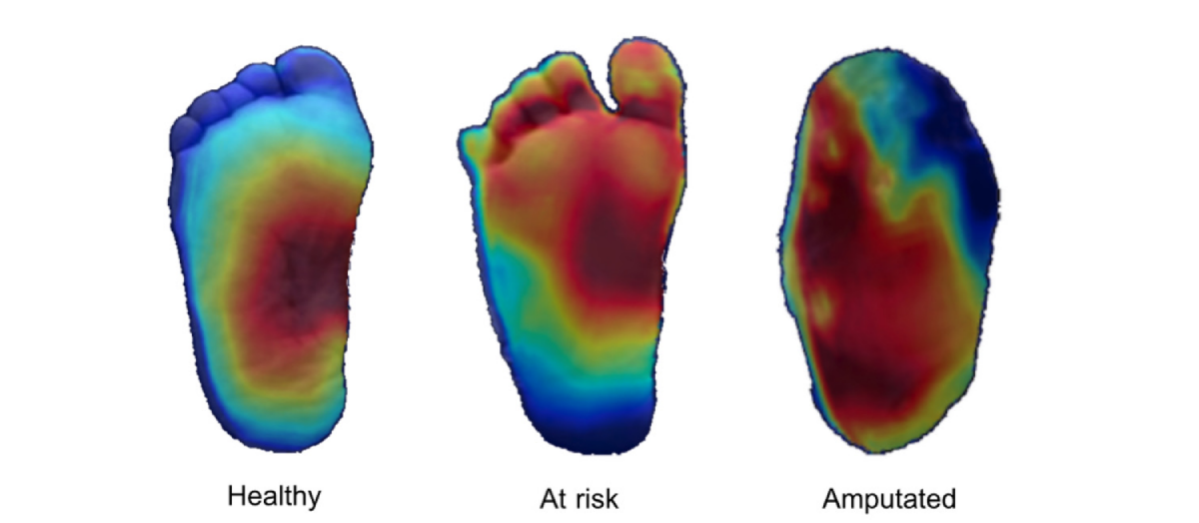
Typical thermal images of the plantar region for healthy, at risk, and amputated patients.

Human medical thermography results from decades of research and development in the performance of infrared imaging, standardization of technique, and clinical protocols for thermal imaging [11,12]. It can visualize diseases not readily detected or monitored through other methods. It is a fast, passive, noncontact, and noninvasive imaging method that has been used in numerous peer-reviewed studies [13]. It is currently used worldwide to screen, detect, and monitor diseases. The American Academy of Thermology (AAT) established guidelines for the use of thermography in the evaluation of patients with diabetes. These guidelines provide recommendations for the use of thermal imaging in the detection and monitoring of diabetic neuropathy, including protocols for image acquisition and interpretation [14]. Figure 1 shows the thermal images of the lower extremities of 2 patients with diabetes.

Human medical thermography has many advantages that could encourage widespread adoption. Thermal imaging is relatively inexpensive, compact, and portable; involves no ionizing radiation; and requires little electric power. Recent technological breakthroughs have transformed large and expensive stationary cameras into portable and inexpensive solutions while maintaining quality imaging [15].

A major limitation of the current state of human medical thermography is that even the most skilled human thermographer can only observe, analyze, and successfully interpret a limited number of thermograms. However, computers can process an image efficiently and extract useful information without tiring. Leveraging artificial intelligence (AI) algorithms, specifically computer vision, can yield findings objectively and minimize interobserver variability. Ongoing progress in software image analysis and reduced reliance on human labor results in faster throughput and centralized processing. This can lead to increased thermographic accuracy and reliability. Nevertheless, computer-aided thermography will require high-level training and experience to ensure quality outcomes.

### DFUs and Thermography

Thermography is helpful for the early detection of abnormalities of the foot by analyzing asymmetries and local temperature changes over time. Assessing temperature differences can enable the early detection of ulcers [16,17]. Peripheral vascular disease is a common complication of diabetes that can result in alterations in blood flow that induce changes in skin temperature. These changes in skin temperature may also indicate tissue damage or inflammation resulting from trauma or excessive pressure. The etiology of these traumas is frequently related to moderate repetitive stress that goes unnoticed due to diabetic neuropathy. The application of thermal imaging for the detection of diabetic foot complications is based on the premise that variations in plantar temperature are associated with these types of complications [4,17-23]. Furthermore, there appears to be a positive correlation between BMI and the risk of diabetic foot complications in patients with type 2 diabetes [24,25].

The rapid development of handheld smartphone-based thermal infrared imagers presents a creative solution for predicting DFUs and monitoring existing ones [26]. To address the lack of thermographers, practical computer vision algorithms are needed to automate the process of image acquisition and analysis. These rapidly expanding, low-cost, and widely available resources can help predict one’s risk of developing foot ulcers, potentially saving limbs and lives.

AI and its applications are increasingly demonstrating promise in the detection and management of DFUs [27-29]. Diabetes foot syndrome, with its lack of early symptoms and significant impact on patients’ quality of life, necessitates the use of AI in timely screening and detection of risk for foot ulcers and possible amputations [28,29].

Studies such as that by Peregrina-Barreto et al [30] have shown the potential of infrared thermography for detecting foot complications in patients with diabetes. Several researchers have demonstrated promising results in the detection of DFUs using machine learning techniques to analyze foot images [31-33]. These studies suggest that AI’s application to data derived from thermal plantar images yields promising results, yet several important gaps remain unaddressed. First, most investigations to date have used specialized infrared cameras in controlled environments, but most have not fully capitalized on low-cost smartphone-based thermal imaging for accessible, point-of-care screening. Preliminary work with smartphone infrared cameras has been limited to small feasibility studies rather than integrated AI screening tools. Second, previous AI models have analyzed foot thermograms as whole images or simple regions without segmenting the foot into angiosomal regions, an approach that could pinpoint localized perfusion deficits or inflammation based on vascular anatomy. Finally, clinical scalability remains an open question: existing studies often involve relatively small samples or single-center data, leading to uncertainty about generalizability to larger, real-world populations with diabetes. This emphasizes the need for a validated solution that addresses these limitations.

To our knowledge, no multicenter study has validated a low-cost smartphone-based thermographic imager paired with an AI algorithm across a large population with diabetes. This study addressed this gap by evaluating Thermal Foot Scan (TFScan), a system that leverages AI techniques combined with thermogram images deployed on a low-cost smartphone-based thermal imager and app.

### Description of the Technology

**Figure 2:**
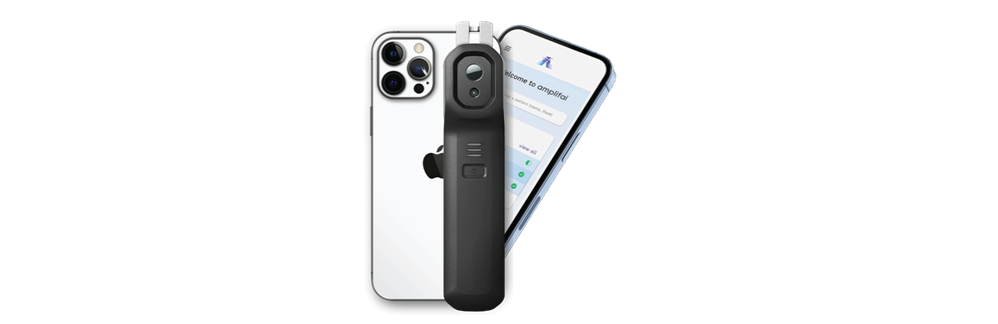
Thermal imaging device.

We created TFScan, a noninvasive system to identify diabetic foot complications at an early stage. We leveraged off-the-shelf thermal cameras compatible with smartphones or tablets to capture detailed thermal images of participants’ feet (Figure 2). We also used AI-based algorithms to perform semantic segmentation and reduce sensor noise in the captured thermograms. The AI models were trained to extract the plantar region as the region of interest and angiosome regions per foot and suppress any background or sensor noise. In addition, the AI was able to detect asymmetric thermal emission of ≥2.2 °C, which can be indicative of pathology in a properly cooled participant [16,34-39].

The system used AI to process images efficiently and extract useful information. It made the findings more objective and minimized interobserver variability. This could lead to faster throughput via centralized cloud-based processing in which samples are anonymized by removing identifiable information from the data, thereby increasing thermographic accuracy and reliability.

The technology analyzes thermal images captured from a specific thermal camera model, the FLIR ONE Edge Pro. We selected the FLIR ONE Edge Pro for its practicality and ease of use in clinical settings. While its resolution is lower than the AAT recommendation of 320 × 240 pixels, several studies have successfully used the FLIR ONE, which has an even lower resolution of 80 × 60 pixels, and concluded that it can effectively capture thermal signals indicative of DFUs [15,37,40,41]. The software identifies temperature variations consistent with inflammation and ulcerative patterns, signaling potential DFUs. The system records, stores, and transmits use events from thermal cameras to a remote storage system.

### Study Objectives

The primary goal of this study was to evaluate the effectiveness of an AI-powered thermographic screening tool in identifying individuals at increased risk of diabetic foot complications through noninvasive, multiregional foot temperature analysis. We also explored the following secondary objectives:

1. To describe the demographic, clinical, and comorbidity profiles of individuals with diabetes enrolled across multiple health care centers in Saudi Arabia
2. To assess the relationship between AI-derived risk classifications and traditional diabetic foot risk factors, including vascular and neuropathic complications
3. To explore temperature-based correlates of AI-predicted risk, including regional foot temperature trends and angiosome-level thermal asymmetry metrics

## Methods

### Study Design and Population

This study was a multicenter cross-sectional analysis of deidentified routine screening data aligned with the Strengthening the Reporting of Observational Studies in Epidemiology reporting guidelines [42]. This study was conducted across diabetes clinics located in Riyadh, Madinah, Qassim, and Al-Baha in the Kingdom of Saudi Arabia. The dataset included participants who were aged >18 years and had been diagnosed with diabetes (type 1 or type 2). Eligibility for screening was assessed through self-report during the routine clinical workflow, where patients who had a visible foot pathology, such as visible ulcers, infections, or amputations, were excluded. In addition, patients who were unable to stand without assistance were excluded due to the higher risk of falling or injuring themselves. Eligible patients were routinely scanned by the nursing team as part of standard diabetic foot screening procedures. Those who met the inclusion criteria proceeded directly to thermal imaging during their clinic visit.

### Data Collection

#### Overview

Data were collected between February 2025 and April 2025 from 1120 patients with diabetes mellitus (types 1 and 2) across multiple diabetic primary care centers. Patients completed a questionnaire that collected demographic information and self-reported clinical data. In addition, the clinical team at each center conducted AI-augmented thermographic imaging of both feet for each participant.

#### Self-Reported Questionnaires

Trained health care professionals collected demographic information through a self-reported questionnaire, including anthropometric measurements (height, weight, and BMI), age, gender, lifestyle factors (smoking status and physical activity level), and diabetes-specific information (type and duration of disease). It also included risk factors such as peripheral artery disease (PAD), neuropathy, diabetes-related complications (retinopathy, chronic kidney disease, and renal failure), foot-specific conditions (onychomycosis, gangrene, and Charcot foot), cardiovascular comorbidities, hypertension, and glycated hemoglobin levels from recent laboratory test results. Additionally, health care professionals documented loss of protective sensation (LOPS) symptoms reported by patients, including unsteadiness in walking, pain and tenderness, numbness, and tingling or prickling sensations, using standardized protocols for comprehensive LOPS assessment.

#### AI-Augmented Thermographic Assessment

Thermal imaging was performed by trained health care professionals who followed a standardized protocol in accordance with the AAT point-of-care guidelines [43]. Patients were acclimatized in a temperature-controlled environment (22-24 °C), after which plantar foot surfaces were captured using a calibrated thermal infrared camera. The captured images were processed through the AI algorithm to extract quantitative thermal parameters, with temperature readings recorded for specific angiosomal regions corresponding to the lateral calcaneal artery, medial calcaneal artery, lateral plantar artery (LPA), and medial plantar artery (MPA), whereas mean foot temperatures were calculated for each foot and temperature asymmetries between corresponding regions of the right and left feet were computed. The AI system analyzed regional thermal distribution patterns for abnormalities, identified hyperthermia and hypothermia in specific angiosomes, quantified temperature asymmetries (with significant asymmetry defined as a ≥2.2 °C difference between corresponding regions), and calculated thermal change index (TCI) scores based on proprietary algorithms.

### Data Quality Assurance

To ensure data integrity and reliability, all clinical assessments were conducted by trained health care professionals following standardized protocols [43], whereas thermal imaging was conducted under controlled environmental conditions to minimize external temperature influences. The AI algorithm underwent regular calibration and validation to maintain accuracy, and all data were entered into a secure electronic database with built-in validation checks to identify outliers and inconsistencies. Random audits of 20% of cases were performed to verify data accuracy throughout the collection process. This comprehensive data collection approach enabled thorough evaluation of the AI-augmented thermography system’s effectiveness in detecting DFU risk while facilitating analysis of correlations among thermal findings, clinical risk factors, and severity scores.

### Ethical Considerations

This study was a secondary analysis of deidentified data obtained during routine clinical procedures and was not part of a prospective research study. All methods were carried out in accordance with relevant guidelines and regulations. The analysis was reviewed and approved by the Ministry of Health Ethics Board, Research Ethics Committee, General Directorate of Research and Studies (H-01-R-009), which waived the requirement for individual informed consent due to the retrospective nature and deidentification of the data. This study adhered to the Declaration of Helsinki and JMIR and Committee on Publication Ethics guidelines on research ethics, privacy, and confidentiality. No additional procedures beyond routine care were performed, and no compensation was provided.

### Experimental Equipment and Procedure

The results of infrared thermography can be influenced by various environmental, individual, and technical factors that affect human skin. To obtain accurate results, the thermal images of the participants in the study were obtained in compliance with the protocols and guidelines set by the AAT [14].

The imaging system records and analyzes thermal asymmetry, temperature distribution, and patterns indicative of pathology. Images were classified based on predefined thresholds for asymmetry (≥2.2 °C) and abnormal thermal patterns. Asymmetric thermal emission of ≥2.2 °C can be indicative of pathology in a properly cooled patient [14].

### Outcome Measures

The primary outcome was the distribution of TFScan-derived thermal risk categories (very low, low, moderate, and high risk) based on clinical risk factors, foot temperature asymmetry, regional thermal patterns, and angiosomal temperatures. Secondary outcomes included (1) the prevalence of thermal abnormalities (hyperthermia, hypothermia, asymmetry of ≥2.2 °C, or abnormal distribution patterns), (2) associations between TFScan risk categories and clinical risk factors (eg, PAD, neuropathy, and retinopathy), (3) comparison of thermal metrics (eg, TCI score, temperature difference, and mean foot and angiosomal temperatures) by clinical condition and risk group, and (4) identification of thermal phenotypes using unsupervised clustering and heat map analysis. These measures evaluated the clinical relevance of TFScan in stratifying diabetic foot risk and characterizing underlying thermal patterns.

### Data Management and Security

All collected data were stored on a password-protected cloud using unique, nonidentifying participant codes. The secure cloud databases are compliant with security standards for access control, data privacy, and encryption requirements. Data were checked for completeness and accuracy, and inconsistencies were resolved through discussion with the research team.

### Statistical Analysis Plan

This was an analytical and exploratory study. We summarized cohort demographics, clinical risk factors, and TFScan-derived thermal variables using appropriate descriptive statistics. Means, SDs, medians, and IQRs were calculated for continuous variables (eg, age, BMI, glycated hemoglobin level, and TCI score), and counts and proportions were used for categorical variables (eg, gender, diabetes type, PAD, and neuropathy). We conducted bivariate analyses to explore associations between TFScan risk categories and clinical variables. Chi-square or Fisher exact tests were used for categorical comparisons, and Kruskal-Wallis tests were used to compare continuous thermal metrics between groups (eg, patients with and without PAD). Results were presented in stratified summary tables by TFScan severity group and clinical condition.

## Results

### Study Population

A total of 1120 individuals with diabetes were enrolled across 4 major regions in Saudi Arabia. The largest proportion of participants was recruited from Riyadh (496/1120, 44.3%), followed by Qassim (305/1120, 27.2%), Madinah (271/1120, 24.2%), and Al-Baha (48/1120, 4.3%), reflecting broad geographic representation. The mean age of the participants was 52.2 (SD 15.85) years, with 73.7% (826/1120) aged >45 years. The cohort was predominantly female (705/1120, 62.9%) and exhibited a mean BMI of 30.62 (SD 6.96) kg/m^2^. The average duration of diabetes was 12.87 (SD 8.57) years, with 63.1% (707/1120) of participants living with diabetes for ≥10 years. Type 2 diabetes accounted for 70% (784/1120) of cases.

### Thermal Imaging Findings

Thermal imaging revealed several distinct abnormalities. Hyperthermia, defined as elevated localized temperature, was observed in 5.9% (66/1120) of participants, whereas hypothermia was present in 2.1% (23/1120). In addition, 7.1% (79/1120) of individuals demonstrated clinically significant temperature asymmetry, defined as a difference of ≥2.2 °C between corresponding angiosomes on the left and right foot. The mean temperature difference between feet was 0.55 (SD 0.56) °C.

**Figure 3:**
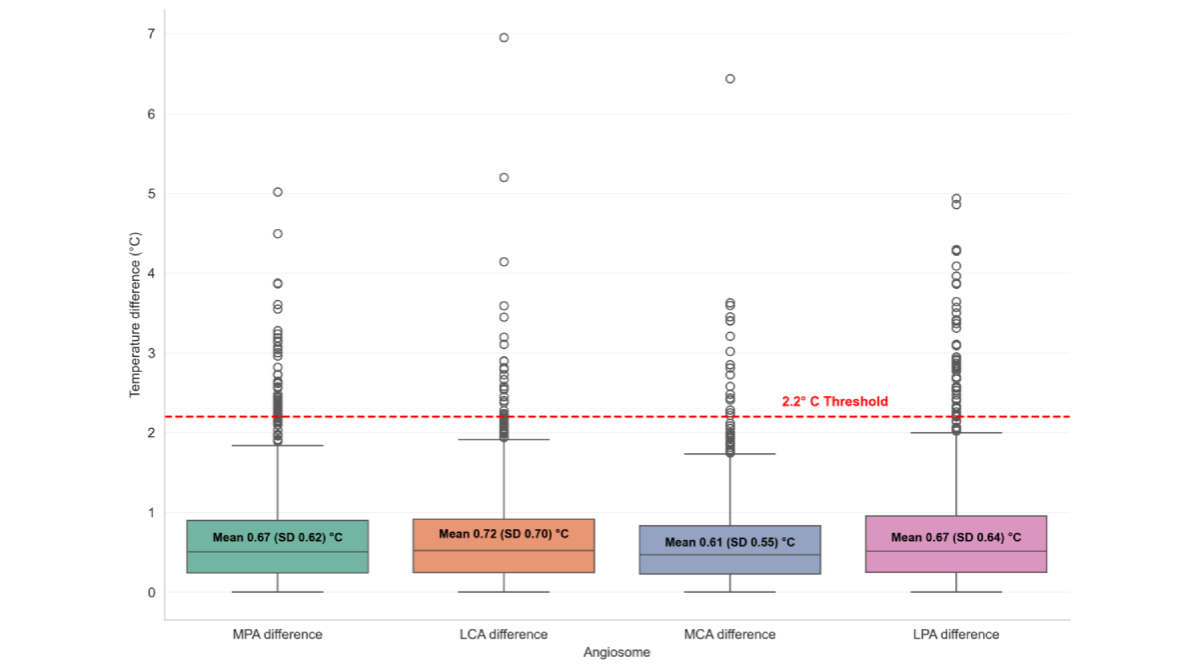
Distribution of angiosome-level temperature differences between feet. LCA: lateral calcaneal artery; LPA: lateral plantar artery; MCA: medial calcaneal artery; MPA: medial plantar artery.

Temperature asymmetries were also examined at the angiosome level. While mean differences were generally under 1 °C, Figure 3 illustrates that a substantial subset of participants exhibited temperature differences exceeding 2 to 3 °C. These outliers may represent individuals at risk of localized inflammation or early tissue compromise not yet detectable through standard clinical examination. Figure 3 shows the distribution of angiosome-level temperature differences between corresponding anatomical regions of the foot.

### Comorbidities

Several chronic complications commonly associated with diabetes were documented in the study cohort. Cardiovascular disease was the most frequently reported comorbidity, affecting 33.3% (373/1120) of participants. Retinopathy was present in 9.6% (108/1120), PAD was present in 2.6% (29/1120), and chronic kidney disease was present in 2% (22/1120). Renal failure was identified in 0.6% (7/1120) of cases, and Charcot foot was diagnosed in 0.1% (1/1120) of the participants. These comorbidities, particularly those affecting vascular and neurological function, are recognized contributors to diabetic foot complications.

### Symptoms of Sensory Loss (LOPS)

Symptoms associated with sensory neuropathy were reported by a substantial proportion of participants. A total of 36.3% (407/1120) experienced at least one symptom consistent with LOPS. The most common symptom was numbness (281/1120, 25.1%), followed by tingling or prickling sensations (185/1120, 16.5%), pain or tenderness (157/1120, 14%), and unsteadiness while walking (82/1120, 7.3%). These sensory abnormalities suggest subclinical neuropathic involvement, which often precedes overt ulceration.

### AI-Based Risk Classification

Using the AI-derived classification system, 13.9% (156/1120) of the participants were categorized as very low risk, 76.8% (860/1120) were categorized as low risk, 4.3% (48/1120) were categorized as moderate risk, and 5% (56/1120) were categorized as high risk. When grouped into binary categories, 90.7% (1016/1120) were considered lower risk (very low+low), whereas 9.3% (104/1120) were flagged as higher risk (moderate+high). The detailed demographic and clinical characteristics of the study population are summarized in Table 1.

**Table 1:** Demographic and clinical characteristics (N=1120).

Characteristic			Values
**Study center, n (%)**			
	Riyadh	496 (44.3)	
	Madinah	271 (24.2)	
	Qassim	305 (27.2)	
	Al-Baha	48 (4.3)	
**Age (years), mean (SD)**			52.2 (15.85)
	<45, n (%)	240 (26.2)	
	45-60, n (%)	446 (39.8)	
	>60, n (%)	380 (33.9)	
**Sex, n (%)**			
	Female	705 (62.9)	
	Male	415 (37.1)	
**Anthropometrics, mean (SD)**			
	BMI (kg/m^2^)	30.62 (6.96)	
	Weight (kg)	78.48 (18.61)	
	Height (cm)	160.25 (9.1)	
**Duration of diabetes (years), mean (SD)**			12.87 (8.57)
	<5, n (%)	215 (19.2)	
	5-10, n (%)	197 (17.6)	
	10-20, n (%)	439 (39.1)	
	≥20, n (%)	269 (24)	
**Diabetes type, n (%)**			
	Type 1	336 (30.0)	
	Type 2	784 (70.0)	
Smoker, n (%)			75 (6.7)
**Physical activity, n (%)**			
	Never	345 (30.8)	
	Rarely	240 (21.4)	
	Once a week	142 (12.7)	
	Several times a week	61 (5.4)	
	Daily	291 (26.0)	
	Missing	41 (3.7)	
**Thermal readings (°C), mean (SD)**			
	Temperature difference (between feet)	0.55 (0.56)	
	TCI^a^	0.67 (0.48)	
	Right foot temperature	27.44 (2.67)	
	Left foot temperature	27.40 (2.64)	
	Right MPA^b^ temperature	28.04 (2.87)	
	Left MPA temperature	27.95 (2.88)	
	Right LCA^c^ temperature	27.57 (2.53)	
	Left LCA temperature	27.63 (2.46)	
	Right MCA^d^ temperature	28.32 (2.40)	
	Left MCA temperature	28.39 (2.38)	
	Right LPA^e^ temperature	27.33 (3.06)	
	Left LPA temperature	27.27 (2.99)	
	MPA difference (between right and left angiosomes)	0.67 (0.64)	
	LCA difference (between right and left angiosomes)	0.67 (0.62)	
	MCA difference (between right and left angiosomes)	0.61 (0.55)	
	LPA difference (between right and left angiosomes)	0.72 (0.70)	
**Thermal abnormalities, n (%)**			
	Hyperthermia (any angiosome)	66 (5.9)	
	Hypothermia (any angiosome)	23 (2.1)	
	Asymmetry of ≥2.2 °C (any foot)	79 (7.1)	
	Hyperthermia in right foot angiosomes	30 (2.7)	
	Hyperthermia in left foot angiosomes	36 (3.2)	
	Hypothermia in right foot angiosomes	12 (1.1)	
	Hypothermia in left foot angiosomes	11 (1.0)	
**Clinical risk factors**			
	HbA_1c_^f^ level (%), mean (SD)	8.23 (2.00)	
	Cardiovascular disease, n (%)	373 (33.3)	
	PAD^g^, n (%)	29 (2.6)	
	Neuropathy, n (%)	49 (4.4)	
	Retinopathy, n (%)	108 (9.6)	
	CKD^h^, n (%)	22 (2.0)	
	Renal failure, n (%)	7 (0.6)	
	History of diabetes of ≥10 y, n (%)	707 (63.1)	
	Charcot foot, n (%)	1 (0.1)	
	Foot deformity, n (%)	21 (1.9)	
**LOPS^i^** **symptoms, n (%)**			
	Any symptom	407 (36.3)	
	Numbness	281 (25.1)	
	Tingling or prickling	185 (16.5)	
	Pain or tenderness	157 (14.0)	
	Unsteady walking	82 (7.3)	
**DFU^j^** **risk category, n (%)**			
	Very low	156 (13.9)	
	Low	860 (76.8)	
	Moderate	48 (4.3)	
	High	56 (5.0)	
**Binary risk classification, n (%)**			
	Lower risk (very low+low)	1016 (90.7)	
	Higher risk (moderate+high)	104 (9.3)	

^a^TCI: thermal change index.

^b^MPA: medial plantar artery.

^c^LCA: lateral calcaneal artery.

^d^MCA: medial calcaneal artery.

^e^LPA: lateral plantar artery.

^f^HbA_1c_: glycated hemoglobin.

^g^PAD: peripheral artery disease.

^h^CKD: chronic kidney disease.

^i^LOPS: loss of protective sensation.

^j^DFU: diabetic foot ulcer.

### Clinical and Protective Sensation Correlates of AI-Predicted Diabetic Foot Risk

To evaluate the clinical correlates of AI-derived risk categories, we compared the prevalence of comorbidities and LOPS symptoms between participants classified as lower risk (very low risk+low risk) versus those in the higher-risk group (moderate risk+high risk).

Several factors were significantly more prevalent among those flagged as higher risk. PAD was observed in 20.2% (21/104) of higher-risk participants compared to just 0.8% (8/1016) in the lower-risk group (*P*<.001). Similarly, cardiovascular disease affected 57.7% (60/104) of the participants in the higher-risk group, a significant difference from 30.8% (313/1016) in the lower-risk cohort (*P*<.001).

**Figure 4:**
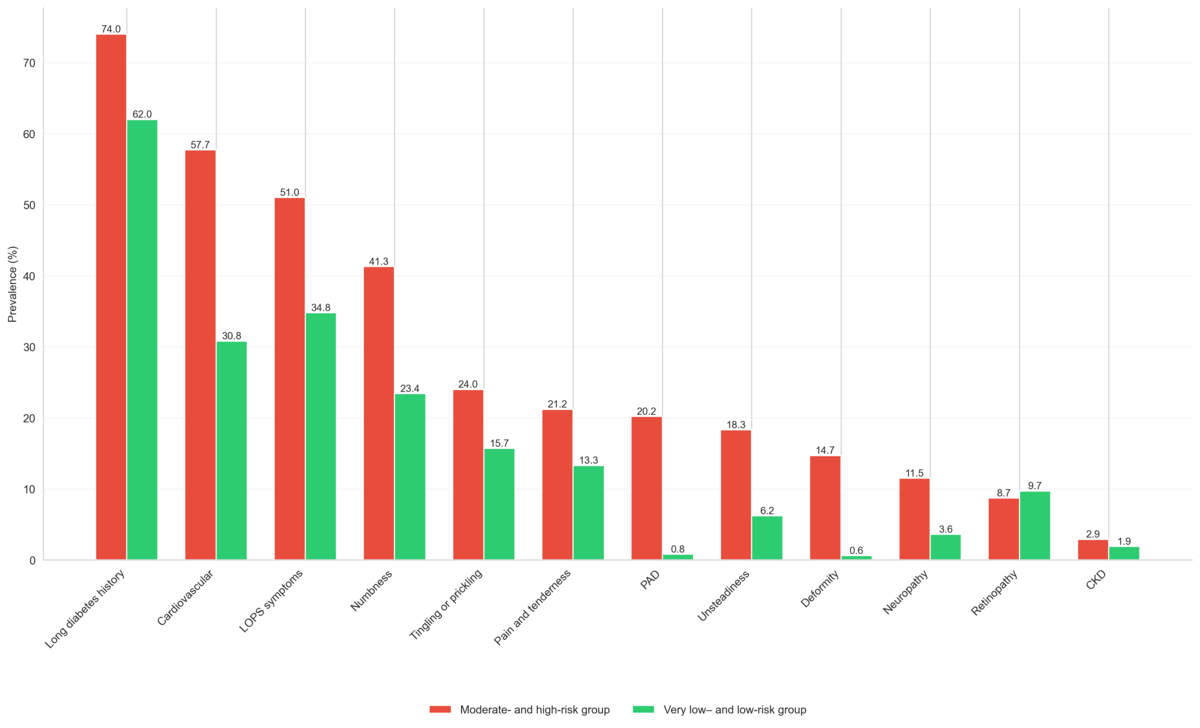
Prevalence of clinical and protective sensation factors by artificial intelligence–classified risk group. CKD: chronic kidney disease; DM: diabetes mellitus; LOPS: loss of protective sensation; PAD: peripheral artery disease.

Neuropathy-related indicators were also more frequent among high-risk individuals. Clinically diagnosed neuropathy was nearly 3 times more common (12/104, 11.5% vs 37/1016, 3.6%; *P*<.001), and LOPS symptoms were present in 51% (53/104) of the participants in the higher-risk group versus 34.8% (354/1016) in the lower-risk group (*P*<.001). Moreover, foot deformities were reported by 14.4% (15/104) of the higher-risk participants compared to only 0.6% (6/1016) of the lower-risk participants (*P*<.001). A longer duration of diabetes (≥10 years) was also more common in the high-risk group (77/104, 74% vs 630/1016, 62%; *P*=.02). These findings support the validity of the AI model in identifying individuals with a higher burden of established diabetic foot risk factors even in the absence of active ulceration. The subgroup comparisons are highlighted in Table 2. These differences are further visualized in Figure 4, which shows a consistent pattern of elevated prevalence of multiple comorbid and neuropathic features among individuals classified as moderate or high risk by the AI model.

**Table 2:** Subgroup comparison by artificial intelligence–classified diabetic foot risk level.

Variable	Moderate and high risk (n=104), n (%)	Very low and low risk (n=1016), n (%)	Prevalence difference (%; 95% CI)	*P* value
PAD^a^	21 (20.2)	8 (0.8)	19.4 (11.7 to 27.1)	<.001
Neuropathy	12 (11.5)	37 (3.6)	7.9 (1.6 to 14.1)	<.001
Retinopathy	9 (8.7)	99 (9.7)	−1.1 (−6.8 to 4.6)	.85
CKD^b^	3 (2.9)	19 (1.9)	1.0 (−2.3 to 4.3)	.73
Diabetes of ≥10 y	77 (74.0)	630 (62.0)	12.0 (3.1 to 21.0)	.02
Charcot foot	1 (1.0)	0 (0.0)	1.0 (−0.9 to 2.8)	.16
Cardiovascular disease	60 (57.7)	313 (30.8)	26.9 (17.0 to 36.8)	<.001
Foot deformity	15 (14.4)	6 (0.6)	14.1 (7.1 to 20.6)	<.001
Any LOPS^c^ symptoms	53 (51.0)	354 (34.8)	16.1 (6.1 to 26.2)	.001
Unsteady walking	19 (18.3)	63 (6.2)	12.1 (4.5 to 19.6)	<.001
Pain or tenderness	22 (21.2)	135 (13.3)	7.9 (−0.3 to 16.0)	.04
Numbness	43 (41.3)	238 (23.4)	17.9 (8.1 to 27.7)	<.001
Tingling or prickling	25 (24.0)	160 (15.7)	8.3 (−0.2 to 16.8)	.04

^a^PAD: peripheral artery disease.

^b^CKD: chronic kidney disease.

^c^LOPS: loss of protective sensation.

### Thermal Pattern Correlates of AI-Predicted Diabetic Foot Risk

To examine how AI-assigned risk categories correspond to objective thermal imaging patterns, we analyzed whole-foot and angiosome-specific temperature metrics across the 4 stratified risk groups.

**Figure 5:**
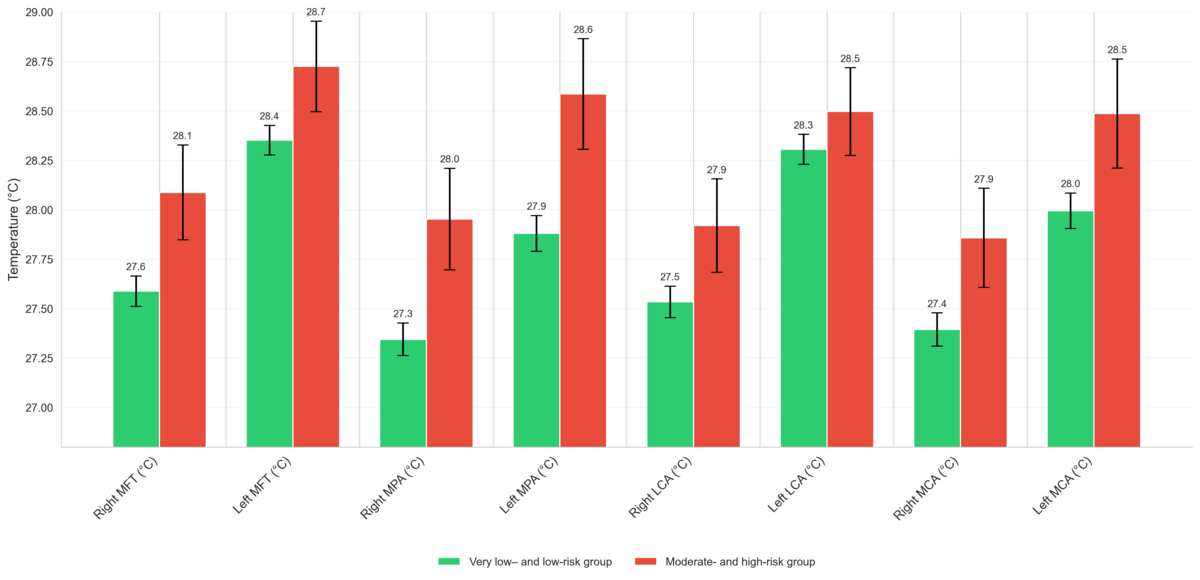
Mean regional foot temperatures across artificial intelligence–predicted risk categories. LCA: lateral calcaneal artery; MCA: medial calcaneal artery; MFT: mean foot temperature; MPA: medial plantar artery.

A clear ascending trend in temperature values was observed from the very low– to moderate-risk categories. For example, mean foot temperatures increased bilaterally from approximately 26.5 °C in the very low–risk group to nearly 28.9 °C in the moderate-risk group. Similarly, temperatures across key angiosomes, including the MPA, lateral calcaneal artery, and medial calcaneal artery, showed rising values across these risk stages. Figure 5 illustrates these dynamic temperature profiles across anatomical regions, reinforcing the value of AI-enhanced thermography in detecting not just risk presence but potentially distinct pathophysiological states within the diabetic foot spectrum.

**Figure 6:**
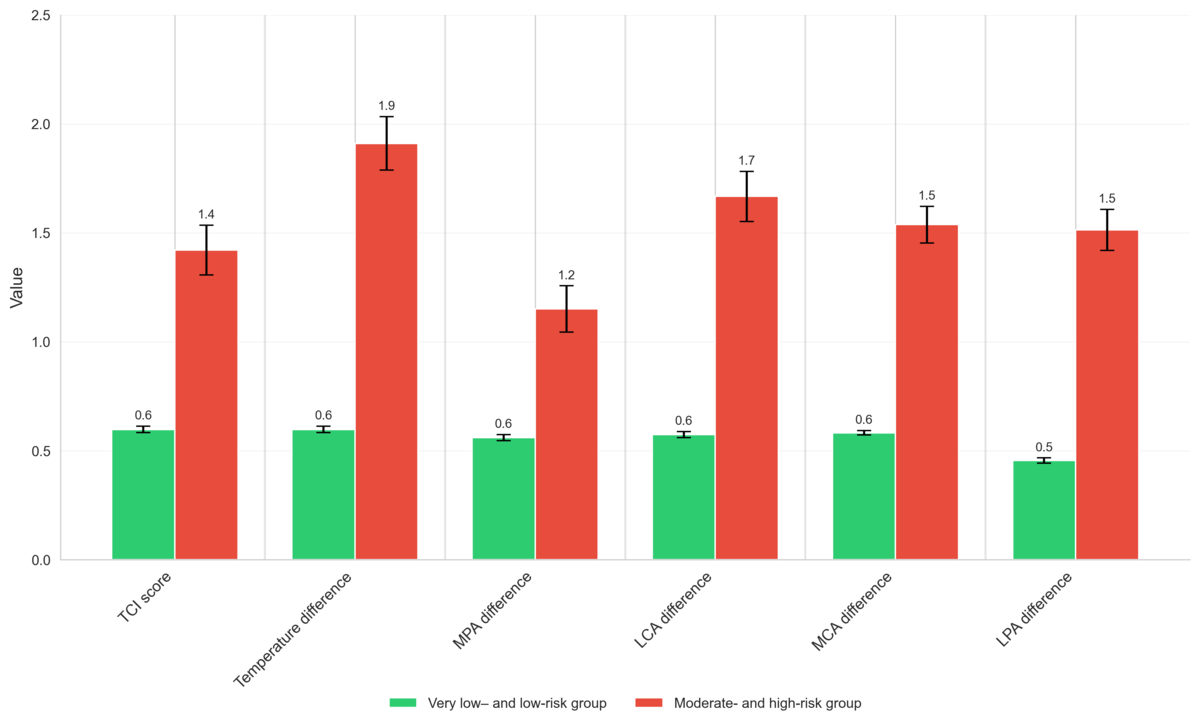
Thermal change index (TCI) and regional temperature asymmetries across artificial intelligence–predicted risk categories. LCA: lateral calcaneal artery; LPA: lateral plantar artery; MCA: medial calcaneal artery; MPA: medial plantar artery.

In addition to absolute temperature values, we examined thermal asymmetry and the TCI, 2 key AI-derived metrics. As shown in Table 3, both TCI and temperature differences across corresponding angiosomes (eg, LPA and MPA) increased substantially with increasing risk categories. Notably, the TCI score increased from 0.58 (SD 0.32) in the very low–risk group to 2.05 (SD 0.74) in the high-risk group, indicating significantly greater thermal irregularity. Similarly, LPA and MPA differences exceeded 2 °C in high-risk participants, far above the thresholds typically associated with early inflammatory changes or preulcerative states. These findings are visualized in Figure 6, which highlights the sharp contrast in asymmetry metrics between lower- and higher-risk categories.

All temperature and asymmetry metrics demonstrated statistically significant differences across AI-predicted risk categories as assessed using the Kruskal-Wallis *H* test (*P*<.005 for all comparisons). These findings support the graded relationship between thermal abnormalities and AI-derived diabetic foot risk. A comprehensive summary of both whole-foot and angiosome-specific mean temperatures, as well as thermal asymmetry metrics across all risk categories, is provided in Table 3.

**Table 3:** Mean foot temperatures and thermal asymmetry metrics by artificial intelligence–predicted diabetic foot risk category.

Variable (°C)	Very low risk, mean (SD)	Low risk, mean (SD)	Moderate risk, mean (SD)	High risk, mean (SD)	*P* value
TCI^a^	0.58 (0.32)	0.58 (0.31)	0.94 (0.55)	2.05 (0.74)	<.001
Temperature difference	0.45 (0.38)	0.46 (0.38)	0.86 (0.65)	2.08 (0.81)	<.001
Right foot temperature	26.53 (2.92)	27.55 (2.61)	28.75 (2.16)	27.09 (2.64)	<.001
Left foot temperature	26.62 (2.86)	27.48 (2.57)	28.93 (2.39)	27.11 (2.53)	<.001
Right MPA^b^ temperature	27.11 (3.12)	28.16 (2.79)	29.42 (2.44)	27.69 (2.87)	<.001
Left MPA temperature	27.10 (3.15)	28.02 (2.80)	29.64 (2.55)	27.68 (2.80)	<.001
Right LCA^c^ temperature	26.68 (2.80)	27.69 (2.46)	28.76 (1.85)	27.20 (2.61)	<.001
Left LCA temperature	26.92 (2.64)	27.71 (2.40)	28.98 (2.19)	27.32 (2.42)	<.001
Right MCA^d^ temperature	27.61 (2.70)	28.43 (2.34)	29.23 (1.83)	27.87 (2.41)	<.001
Left MCA temperature	27.70 (2.66)	28.47 (2.31)	29.63 (1.91)	27.95 (2.40)	<.001
MPA difference (right–left)^e^	0.56 (0.44)	0.58 (0.44)	0.92 (0.74)	2.30 (1.09)	<.001
LCA difference (right–left)	0.63 (0.50)	0.59 (0.46)	0.97 (0.80)	1.81 (1.29)	<.001
MCA difference (right–left)	0.54 (0.44)	0.56 (0.43)	0.77 (0.73)	1.48 (1.22)	<.001
LPA^f^ difference (right–left)	0.59 (0.44)	0.60 (0.47)	1.09 (0.83)	2.62 (1.12)	<.001

^a^TCI: thermal change index.

^b^MPA: medial plantar artery.

^c^LCA: lateral calcaneal artery.

^d^MCA: medial calcaneal artery.

^e^Between the right and left angiosomes.

^f^LPA: lateral plantar artery.

## Discussion

### Principal Findings

This multicenter, cross-sectional study evaluated the clinical utility of TFScan, an AI-assisted thermographic tool, in detecting thermal abnormalities indicative of early diabetic foot complications. Our results demonstrate that TFScan can stratify patients with diabetes into clinically meaningful risk categories. The distinct thermographic profiles across risk strata and the strong correlation with established clinical risk factors such as PAD, cardiovascular disease, neuropathy, and LOPS support the biological plausibility of TFScan’s classifications. Additionally, patients classified as moderate or high risk by the AI algorithm exhibited significantly elevated temperature asymmetry, higher TCI scores, and greater deviations in angiosomal temperatures. These findings suggest that thermal imaging, when integrated with AI algorithms, may offer a scalable and noninvasive approach for early diabetic foot screening in primary care settings.

### Comparison With Existing Literature

The significant differences in comorbidity prevalence between risk groups affirm the clinical relevance of TFScan’s classifications. PAD and cardiovascular disease were markedly more common in the higher-risk group, consistent with the vascular underpinnings of diabetic foot complications. Neuropathy and LOPS symptoms, including numbness and unsteadiness, were also elevated among high-risk individuals, in line with previous findings that associate sensory loss with foot ulceration risk [4]. However, we extend prior work by evaluating a smartphone‑based system across more than 1100 participants.

Mechanistically, hyperthermic patterns likely reflect neuropathic inflammation, whereas marked hypothermia and attenuated angiosomal temperatures may signal ischemic vascular disease. TFScan revealed progressive increases in mean foot and angiosome temperatures from the very low– to moderate-risk groups, followed by a decline in the high-risk category, likely due to underlying hypoperfusion. This nonlinear pattern highlights the algorithm’s sensitivity to both hyperthermic inflammation and hypothermic perfusion deficits and, therefore, its ability to detect both neuropathic and vascular complications by integrating both absolute temperatures and asymmetries. The pronounced temperature asymmetry in high-risk participants, particularly in the MPA and LPA angiosomes, aligns with previous reports that asymmetry of ≥2.2 °C is predictive of preulcerative conditions [4,17-23]. Our TCI score results further reinforce thermal irregularity as a viable biomarker.

### Clinical Implications

These results suggest that TFScan may serve as a practical adjunct to existing diabetic foot screening methods, especially in resource-limited or high-volume clinical settings, where it could be deployed during routine visits to flag individuals needing vascular assessment or podiatry referral. Its portability, automation, noninvasiveness, and objectivity offer significant advantages over subjective assessments such as the monofilament test, which often suffers from interrater variability and limited sensitivity to early neuropathy or vascular dysfunction. By identifying thermal risk patterns early, TFScan may facilitate timely referral, preventive interventions, and more personalized diabetic foot care. These features suggest strong potential for integration into routine diabetes care, particularly in primary and remote care settings where advanced diagnostics may not be available. Nevertheless, integration would require training of staff, calibration of devices, and clear referral pathways.

### Strengths and Limitations

This study benefited from a large, multicenter design and standardized imaging protocols, enhancing the generalizability of our findings. The use of AI-based image analysis minimized observer bias and enabled quantitative assessment of subtle thermal patterns. Additionally, our inclusion of both clinical and thermographic variables allowed for comprehensive profiling of risk.

Despite these advantages, several limitations must be acknowledged. First, the cross-sectional design precludes causal inference or temporal assessment of ulcer development. Longitudinal follow-up is necessary to determine whether thermal abnormalities predict future foot complications. Second, self‑reported data on comorbidities and LOPS symptoms may be affected by recall bias. Moreover, the absence of independent blinding and the relatively low prevalence of advanced complications (eg, PAD and renal failure) in the sample limited statistical power for some comparisons. Finally, while care was taken to standardize image acquisition protocols under controlled environmental conditions, unmeasured confounders (such as prescreening physical activity, ambient room temperature, or skin moisture) may have influenced participants’ skin and, therefore, the thermal readings.

These strengths and limitations outline both the promise and the current boundaries of AI-augmented thermographic screening. Future work should build on this foundation through prospective validation studies and technical refinement of the imaging and analysis pipeline.

### Future Directions

Future research should evaluate the prognostic utility of TFScan classifications in predicting DFU development and other clinical end points. Longitudinal cohort studies or pragmatic trials integrating TFScan into routine care pathways would be essential to assess real-world effectiveness. Moreover, to address current methodological limitations, subsequent studies should replace self-reported comorbidity and LOPS data with objective measures such as electronic health record linkage and clinician-verified neuropathy or vascular assessments and incorporate independent, blinded outcome assessment to reduce the risk of bias.

Given the low prevalence of advanced complications in this cohort, validation should include enriched and diverse recruitment to ensure adequate event rates, enable subgroup analyses, and strengthen generalizability. Furthermore, efforts should be made to refine the algorithm to incorporate more granular thermal features (eg, localized angiosomal gradients) and explore integration with additional clinical variables or sensor data. Technical refinement should also prioritize robust image acquisition and normalization, for example, the capture of preimaging activity (eg, step count) and skin moisture to allow for covariate adjustment. Finally, implementation science frameworks are needed to study the barriers, facilitators, and cost-effectiveness of TFScan integration at scale.

### Conclusions

In this multicenter screening study, AI-assisted thermographic imaging using TFScan effectively stratified patients with diabetes into clinically meaningful risk categories for foot complications. Higher TFScan risk aligns with greater burdens of PAD, cardiovascular disease, and neuropathy and with graded abnormalities in temperature asymmetry, angiosome-level differences, and the TCI, supporting construct validity. However, the cross-sectional design, partial reliance on self-report, and low prevalence of advanced complications may limit causal inference. These findings support the potential role of TFScan as a scalable, noninvasive tool for early identification of patients at risk of diabetic foot complications, meriting further longitudinal validation.

## Abbreviations

AAT: American Academy of Thermology
AI: artificial intelligence
DFU: diabetic foot ulcer
LOPS: loss of protective sensation
LPA: lateral plantar artery
MPA: medial plantar artery
PAD: peripheral artery disease
TCI: thermal change index
TFScan: Thermal Foot Scan

## References

[1] Sun H, Saeedi P, Karuranga S, Pinkepank M, Ogurtsova K, Duncan BB, Stein C, Basit A, Chan JC, Claude Mbanya J, Pavkov ME, Ramachandaran A, Wild SH, James S, Herman WH, Zhang P, Bommer C, Kuo S, Boyko EJ, Magliano DJ (2023). Erratum to "IDF diabetes atlas: global, regional and country-level diabetes prevalence estimates for 2021 and projections for 2045" [Diabetes Res. Clin. Pract. 183 (2022) 109119]. Diabetes Res Clin Pract.

[2] Armstrong DG, Swerdlow MA, Armstrong AA, Conte MS, Padula WV, Bus SA (2020). Five year mortality and direct costs of care for people with diabetic foot complications are comparable to cancer. J Foot Ankle Res.

[3] Armstrong DG, Boulton AJ, Bus SA (2017). Diabetic foot ulcers and their recurrence. N Engl J Med.

[4] Ilo A, Romsi P, Mäkelä J (2020). Infrared thermography and vascular disorders in diabetic feet. J Diabetes Sci Technol.

[5] Wang F, Zhang J, Yu J, Liu S, Zhang R, Ma X, Yang Y, Wang P (2017). Diagnostic accuracy of monofilament tests for detecting diabetic peripheral neuropathy: a systematic review and meta-analysis. J Diabetes Res.

[6] Bhasin N, Scott DJ (2007). Ankle Brachial Pressure Index: identifying cardiovascular risk and improving diagnostic accuracy. J R Soc Med.

[7] Casey SL, Lanting SM, Chuter VH (2020). The ankle brachial index in people with and without diabetes: intra-tester reliability. J Foot Ankle Res.

[8] Eiberg JP, Grønvall Rasmussen JB, Hansen MA, Schroeder TV (2010). Duplex ultrasound scanning of peripheral arterial disease of the lower limb. Eur J Vasc Endovasc Surg.

[9] Collins R, Cranny G, Burch J, Aguiar-Ibáñez R, Craig D, Wright K, Berry E, Gough M, Kleijnen J, Westwood M (2007). A systematic review of duplex ultrasound, magnetic resonance angiography and computed tomography angiography for the diagnosis and assessment of symptomatic, lower limb peripheral arterial disease. Health Technol Assess.

[10] Amin N, Doupis J (2016). Diabetic foot disease: from the evaluation of the "foot at risk" to the novel diabetic ulcer treatment modalities. World J Diabetes.

[11] Campbell JS, Mead MN (2022). Human Medical Thermography.

[12] Serbu G (2009). Infrared imaging of the diabetic foot. Proceedings of the 10th Conference on 2009 InfraMation.

[13] Lahiri BB, Bagavathiappan S, Jayakumar T, Philip J (2012). Medical applications of infrared thermography: a review. Infrared Phys Technol.

[14] Schwartz RG, Brioschi M, O’Young B, Getson P, Bernton T, Brioschi M, Zhang HY, Schakaraschwilli G, Terzella M, Habibi B (2022). The American Academy of Thermology Guidelines for Neuro-Musculoskeletal 2021: infrared medical thermology and sympathetic skin response (SSR) studies. Pan Am J Med Thermol.

[15] van Doremalen RF, van Netten JJ, van Baal JG, Vollenbroek-Hutten MM, van der Heijden F (2019). Validation of low-cost smartphone-based thermal camera for diabetic foot assessment. Diabetes Res Clin Pract.

[16] Ilo A (2020). Infrared thermography in vascular disorders - screening and follow-up. University of Oulu.

[17] Nagase T, Sanada H, Takehara K, Oe M, Iizaka S, Ohashi Y, Oba M, Kadowaki T, Nakagami G (2011). Variations of plantar thermographic patterns in normal controls and non-ulcer diabetic patients: novel classification using angiosome concept. J Plast Reconstr Aesthet Surg.

[18] de Deus Passos M, da Rocha AF (2022). Evaluation of infrared thermography with a portable camera as a diagnostic tool for peripheral arterial disease of the lower limbs compared with color Doppler ultrasonography. Arch Med Sci Atheroscler Dis.

[19] Zhou Q, Qian Z, Wu J, Liu J, Ren L, Ren L (2021). Early diagnosis of diabetic peripheral neuropathy based on infrared thermal imaging technology. Diabetes Metab Res Rev.

[20] Hernandez-Contreras DA, Peregrina-Barreto H, Rangel-Magdaleno JDJ, Renero-Carrillo FJ (2019). Plantar thermogram database for the study of diabetic foot complications. IEEE Access.

[21] Sudha BG, Umadevi V, Shivaram JM, Sikkandar MY, Al Amoudi A, Chaluvanarayana HC (2018). Statistical analysis of surface temperature distribution pattern in plantar foot of healthy and diabetic subjects using thermography. Proceedings of the 2018 International Conference on Communication and Signal Processing.

[22] Bagavathiappan S, Philip J, Jayakumar T, Raj B, Rao PN, Varalakshmi M, Mohan V (2010). Correlation between plantar foot temperature and diabetic neuropathy: a case study by using an infrared thermal imaging technique. J Diabetes Sci Technol.

[23] Papanas N, Papatheodorou K, Papazoglou D, Kotsiou S, Maltezos E (2010). Association between foot temperature and sudomotor dysfunction in type 2 diabetes. J Diabetes Sci Technol.

[24] Renero-Carrillo FJ (2017). The thermoregulation of healthy individuals, overweight–obese, and diabetic from the plantar skin thermogram: a clue to predict the diabetic foot. Diabet Foot Ankle.

[25] Neves EB, Almeida AJ, Rosa C, Vilaca-Alves J, Reis VM, Mendes R (2015). Anthropometric profile and diabetic foot risk: a cross-sectional study using thermography. Annu Int Conf IEEE Eng Med Biol Soc.

[26] Basatneh R, Najafi B, Armstrong DG (2018). Health sensors, smart home devices, and the internet of medical things: an opportunity for dramatic improvement in care for the lower extremity complications of diabetes. J Diabetes Sci Technol.

[27] Alwashmi MF, Alghali M, AlMogbel A, Alwabel AA, Alhomod AS, Almaghlouth I, Temsah M, Jamal A (2025). The use of AI-powered thermography to detect early plantar thermal abnormalities in patients with diabetes: cross-sectional observational study. JMIR Diabetes.

[28] Chemello G, Salvatori B, Morettini M, Tura A (2022). Artificial intelligence methodologies applied to technologies for screening, diagnosis and care of the diabetic foot: a narrative review. Biosensors (Basel).

[29] Kaselimi M, Protopapadakis E, Doulamis A, Doulamis N (2022). A review of non-invasive sensors and artificial intelligence models for diabetic foot monitoring. Front Physiol.

[30] Peregrina-Barreto H, Morales-Hernandez LA, Rangel-Magdaleno JJ, Avina-Cervantes JG, Ramirez-Cortes JM, Morales-Caporal R (2014). Quantitative estimation of temperature variations in plantar angiosomes: a study case for diabetic foot. Comput Math Methods Med.

[31] Cruz-Vega I, Hernandez-Contreras D, Peregrina-Barreto H, Rangel-Magdaleno JD, Ramirez-Cortes JM (2020). Deep learning classification for diabetic foot thermograms. Sensors (Basel).

[32] Goyal M, Reeves ND, Rajbhandari S, Ahmad N, Wang C, Yap MH (2020). Recognition of ischaemia and infection in diabetic foot ulcers: dataset and techniques. Comput Biol Med.

[33] Khandakar A, Chowdhury ME, Ibne Reaz MB, Md Ali SH, Hasan MA, Kiranyaz S, Rahman T, Alfkey R, Bakar AA, Malik RA (2021). A machine learning model for early detection of diabetic foot using thermogram images. Comput Biol Med.

[34] Armstrong DG, Lavery LA (1996). Monitoring neuropathic ulcer healing with infrared dermal thermometry. J Foot Ankle Surg.

[35] Houghton VJ, Bower VM, Chant DC (2013). Is an increase in skin temperature predictive of neuropathic foot ulceration in people with diabetes? A systematic review and meta-analysis. J Foot Ankle Res.

[36] van Netten JJ, van Baal JG, Liu C, van der Heijden F, Bus SA (2013). Infrared thermal imaging for automated detection of diabetic foot complications. J Diabetes Sci Technol.

[37] Vilcahuaman L, Canals R, Canals R, Zequera M, Wilches C, Arista MT, Torres L, Arbañil H (2015). Automatic analysis of plantar foot thermal images in at-risk type II diabetes by using an infrared camera. Proceedings of the 2015 World Conference on Medical Physics and Biomedical Engineering.

[38] Fraiwan L, AlKhodari M, Ninan J, Mustafa B, Saleh A, Ghazal M (2017). Diabetic foot ulcer mobile detection system using smart phone thermal camera: a feasibility study. Biomed Eng Online.

[39] Schaper NC, van Netten JJ, Apelqvist J, Bus SA, Fitridge R, Game F, Monteiro-Soares M, Senneville E (2024). Practical guidelines on the prevention and management of diabetes-related foot disease (IWGDF 2023 update). Diabetes Metab Res Rev.

[40] Murphy CA, Laforet K, Da Rosa P, Tabamo F, Woodbury MG (2012). Reliability and predictive validity of inlow's 60-second diabetic foot screen tool. Adv Skin Wound Care.

[41] Qin Q, Nakagami G, Ohashi Y, Dai M, Sanada H, Oe M (2022). Development of a self-monitoring tool for diabetic foot prevention using smartphone-based thermography: plantar thermal pattern changes and usability in the home environment. Drug Discov Ther.

[42] von Elm E, Altman DG, Egger M, Pocock SJ, Gøtzsche PC, Vandenbroucke JP (2008). The strengthening the reporting of observational studies in epidemiology (STROBE) statement: guidelines for reporting observational studies. J Clin Epidemiol.

[43] Guidelines for point of care medical thermography. American Academy of Thermology.

